# Comparison of three nudge interventions (priming, default option, and perceived variety) to promote vegetable consumption in a self-service buffet setting

**DOI:** 10.1371/journal.pone.0176028

**Published:** 2017-05-31

**Authors:** Rasmus Friis, Laurits Rohden Skov, Annemarie Olsen, Katherine Marie Appleton, Laure Saulais, Caterina Dinnella, Heather Hartwell, Laurence Depezay, Erminio Monteleone, Agnès Giboreau, Federico J. A. Perez-Cueto

**Affiliations:** 1 Department of Food Science, University of Copenhagen, Frederiksberg C, Denmark; 2 Institute for Planning & Development, Aalborg University, Copenhagen SV, Denmark; 3 Research Centre for Behaviour Change, Department of Psychology, Faculty of Science and Technology, Bournemouth University, Poole, Dorset, United Kingdom; 4 Centre for Food and Hospitality Research, Institut Paul Bocuse, Chateau du vivier, Ecully Cedex, France; 5 UMR GAEL, CNRS, INPG, INRA, Université Grenoble-Alpes, Saint Martin d'Hères, France; 6 Department of Management of Agricultural, Food and Forestry Systems, University of Florence, Florence, Italy; 7 School of Tourism, Foodservice and Applied Nutrition Research Group & Health and Wellbeing, Faculty of Management, Bournemouth University, Bournemouth, United Kingdom; 8 BONDUELLE Corporate Research & Communication, Food & Behaviours department, Fondation Louis Bonduelle, Villeneuve D’Ascq, France; Leibniz-Institut fur Pflanzengenetik und Kulturpflanzenforschung Gatersleben, GERMANY

## Abstract

**Background:**

Dietary choices in out-of-home eating are key for individual as well as for public health. These dietary choices are caused by a wide array of determinants, one of which is automatic decision-making. Nudging is attracting considerable interest due to its understanding and application of heuristic biases among consumers. The aim of this study is to test and compare three nudges in promoting vegetable consumption among test persons in a food lab-based experiment.

**Methods:**

The initial sample consisted of 88 participants recruited in Copenhagen, Denmark. Each study participant was randomly assigned to one of the three experiments: priming, default and perceived variety. The priming arm of the experiment consisted of creating a leafy environment with green plants and an odour of herbs. In the default arm of the experiment, the salad was pre-portioned into a bowl containing 200g of vegetables. The third experiment divided the pre-mixed salad into each of its components, to increase the visual variety of vegetables, yet not providing an actual increase in items. Each individual was partaking twice thus serving as her/his own control, randomly assigned to start with control or experimental setting.

**Results:**

The default experiment successfully increased the energy intake from vegetables among the study participants (124 kcal vs. 90 kcal in control, p<0.01). Both the priming condition and perceived variety reduced the total energy intake among the study participants (169 kcal, p<0.01 and 124 kcal, p<0.01, respectively), mainly through a decrease in the meat-based meal component.

**Conclusions:**

Considerable progress has been made with regard to understanding the use of nudging in promoting a healthier meal composition, including increasing vegetable intake. This study suggests that the nature of a nudge-based intervention can have different effects, whether it is increasing intake of healthy components, or limiting intake of unhealthy meal components. This work has demonstrated that consumer behaviour can be influenced without restricting or providing incentives for behaviour change. The present findings have promising application to the foodservice sector.

## Background

Poor dietary choices, such as overconsumption of energy-dense foods and low intakes of fruit and vegetables, have been associated with an increased risk of non-communicable diseases (NCDs) such as, obesity, type II diabetes and ischemic heart diseases. According to the World Health Organization, e.g. NCDs account for 90% of all disease-related deaths in Denmark [1], and it is expected to increase by 13% by the year 2020 [2]. A diet composed of more plant origin (fruits and vegetables) can decrease the incidence of NCD [3]. The current public health challenge is to replace high energy dense foods with fruits and vegetables in order to reduce the energy content of the diet [4]. The majority of the Danish population have knowledge of the recommendations, which have been in place for the last 17 years, supported by strong health campaigning [5]. They also have the economic and environmental ability to follow them. Yet, there is a gap between this knowledge and actual behavioural action [5], and the intake of fruits and vegetables neither matches the recommendations among men nor among women in Denmark [6].

Out-of-home eating is widely considered a part of the modern lifestyle and the public food environment has a central role to play in providing a sense of quality in diets and ensuring public health. Yet, research suggests that out-of-home eating is linked to more energy dense meals [7, 8], larger portion sizes [9, 10], and poor nutritional intake [11, 12]. Canteen and self-service buffets provide customers the opportunity to assemble a meal according to their own preference. However, food choice behaviours are highly complex and determined by several interrelating factors such as availability, liking and quality. Furthermore, eating is also a source of pleasure and comfort, and reflects our personal and cultural characteristics, social status and relationships.

After decades of addressing unhealthy dietary behaviours through information campaigns, legislation and education, the public health sector now suggests targeting the food environment in order to promote desirable food choices [13]. Recent research in consumer science acknowledges that a food decision depends partly upon how the choice is presented at point-of-purchase [14–17], as well as the previously mentioned determinants. Behavioural economic theories of decision-making suggest that food choices can be nudged towards healthier meal selections by changing the choice task [18, 19]. This is done by making a choice architectural change that alters people’s behaviour in a predictable way without forbidding any options or significantly changing their economic incentives [19]. As such, a person’s behaviour may be altered without having to impose or force them into actively engaging in healthier choices. One approach of nudging is priming, that is to say, providing cues in the environment that unconsciously drive decisions. For instance, visual stimuli and odours of food successfully increase hunger by influencing dopamine’s action in certain regions of the brain [20]. Likewise, ambience affects food intake in that smell, light as well as colour have substantial impact on food choice [21]. A second approach is offering a default choice. The default option recommends a course of action, which requires no or little reflection, where consumers will ‘go with the flow’ of pre-set options such as type of food or the choice of clearing the plate where the consumer will consume what the plate holds [22, 23]. Notably, studies show that portion size (which constitutes a default quantity) is a significant determinant of food intake [9, 24–27]. A third approach is to increase the perceived variety of healthy food choices. It has been shown that variety is valued by consumers, as a variety of options increases the chance of matching optimal consumer preference [28]. Meengs and colleagues found that proposing a variety of three different vegetables (carrots, broccoli and snap peas) increased the consumption compared to serving only one of the vegetable items [29]. Similarly, it has been demonstrated across studies that increased variety, or increase in perceived variety, can lead to an increase in food intake [17, 29–33]. However, extending the (perceived) amount of options may also increase the cognitive burden of evaluating opportunities [28], depending on the extent and the nature of the variety proposed [34].

The objective within the present study is to investigate whether nudging can be used to influence vegetable intake at a self-service buffet. In particular the aim was to investigate and compare the efficiency of priming, using default choice options, and increasing perceived variety.

## Methods

The study had an experimental cross-over design with three treatments corresponding to the three Nudge strategies (priming, default and perceived variety) and a control serving. The participants were randomized to one of the three nudge strategies and a control serving. The experiment was conducted at the FoodScape Lab at Aalborg University, Copenhagen, reproducing the settings of a self-serving buffet [35]. To achieve as natural a lunch-situation as possible, participants were not informed of the overall study purpose and food served was similar to what you would normally find in the campus canteen. In total there were 11 test days; the three experiments were dispersed over six days (two for each experimental setting) and there were five control days. The order of the sessions were randomized. Each participant attended one experimental and one control session. The food was provided free of charge, but no further incentives were used.

### Recruitment

Participants were recruited through advertisement posters, social media and through the website forsoegsperson.dk (trial volunteers website). Recruitment posters were set up at train stations, workplaces, grocery shops and Universities in Frederiksberg, Valby, Sydhavnen and Nørrebro in Copenhagen, while on social media a word-of-mouth strategy was employed [36]. The inclusion criteria were: men and women between 18 and 55 years of age. Participants were excluded if they were on a special diet (weight lost/gain, religious diet and vegetarian/vegan) or with the intention of changing smoking or exercising habits, and/or planning to be or already being pregnant. After recruitment, participants were randomly allocated to either start with their control setting or experimental setting in a balanced manner.

### Measures

A web-based questionnaire was distributed among recruited participants prior to their first eating session. The questionnaire contained questions about gender, age, educational achievement and self-reported height (cm) and weight (kg). The latter were used to calculate Body Mass Index (BMI) [37]. Educational achievement was grouped into one of three: Educational level I, which included people who had finished up to 3 years at University or less; Educational level II, included people who had finished a college bachelors degree (3–4 years) and lastly; Educational level III, which included people who have finished University at Masters level or Ph.D. (>4 years). The web-based questionnaire also included a short self-administered food frequency questionnaire, regarding weekly consumption of salad, cooked vegetables or veggies for snacking, with the following options: never/seldom, once per month, 2–3 times per month, 1–2 times per week, 3–4 times per week, 5–6 times per week, once a day, 2 or more times a day. It included also a measure of self-perceived fruit & vegetable intake, estimated on a perceived daily consumption, calculated on the basis of given portion weighs (1 carrot 100g, 1 tomato 60g, ½ paprika 40 g and ¼ cucumber 75g) and self-perceived health (very good, good, tolerable, bad, very bad). The questionnaire was in Danish and constructed with pre-tested and validated parts from the Danish KRAM-survey, the Danish OPUS study and the American NHANES survey [38, 39].

Participants furthermore filled out a questionnaire on appetite just before and just after each eating session. They scored their appetite on a 10-point Likert scale anchored from “I am not at all” (score: 1) to “I have never been more” (score: 10). Participants gave two scores before eating: I) "how hungry are you?" (10 points scale, from not at all hungry = 1 to I have never been so hungry = 10) II) "how much do you think you can eat?" (10 points scale, from nothing at all = 1 to a lot = 10) and two after eating: I) can you eat more? (10 points scale, from nothing at all = 1 to a lot = 10) II) How satisfied are you (I am not at all satisfied = 1 to I can’t eat anything more = 10?

### Food & serving equipment

The experiment was conducted during a typical Danish lunch period from 11.30am to 1pm. There was no maximum set for the lunch duration of the study, but the observed average dining session lasted for approximately 30 minutes. Food was served at an Intelligent Buffet (iBuffet) in a FoodScape Lab that was described elsewhere [35]. This installation and its equipment allow exact measurement in grams and automatic register of each food item that the participants took from the iBuffet, after they scan a wristband used for identification. Leftovers on the plate were weighed afterwards to determine exactly how much of each food item by the participants. Foods were: white boiled and salted rice, chilli con carne (kidney beans, tomato, minced beef, onion, garlic, chilli, cumin, dark chocolate) and white salad (white cabbage, cauliflower, orange, rocket salad, pea sprouts, olive oil, honey and apple cider vinegar); which were served in 5L square bowls with sharp edges. Rice and chilli con carne were served using a 1 dl serving spoon. Red salad (grilled peppers, zucchini, watercress and vinegar) was also offered and served in three-litre square bowls. For the white and red salad the serving was with tongs. Salsa (tomato, onion, garlic, parsley and jalapenos) was served in a 0.8L white porcelain jar with 0.2 dl ladle. Foods were served ad libitum and continuously replenished. Participants were allowed to help themselves at the buffet as many times as they wanted. Plate size was 24cm in diameter, 25cl water glass and 1.4L transparent water carafe. All tableware was placed at on a separate cutlery table. All recipes are available as supplementary materials.

**Fig 1:**
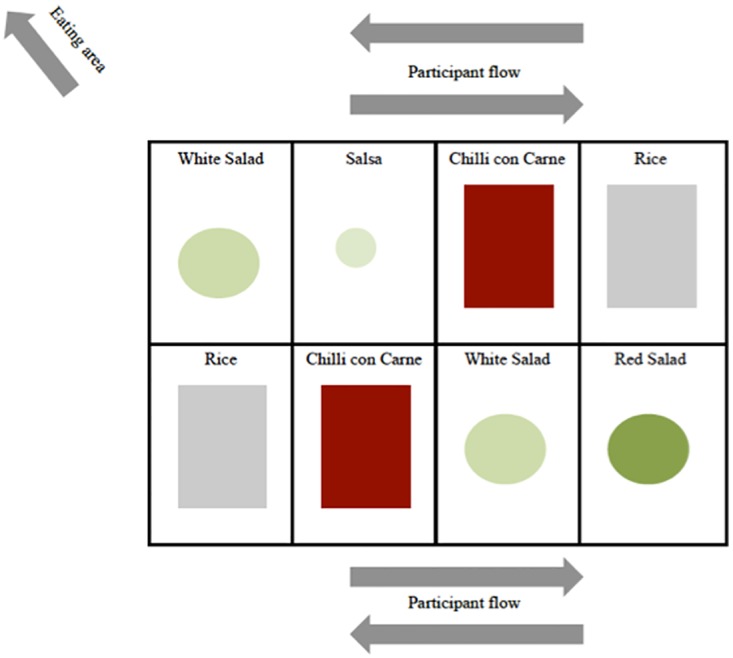
Visual presentation of the control serving.

The iBuffet had eight serving surfaces, four in each side (See Fig 1) with a mix of cold and warm surfaces. Participants had to check-in at each station where they took anything from the buffet.

### Experimental design

All tables, chairs, iBuffet and observation cameras had the same setup during data collection for all servings to ensure coherence and limit bias. The experimental settings were only changed as explicitly explained in the following paragraphs.

#### Control

The food environment was not manipulated. The buffet was organised for self-serving, and each dish component was presented in a large bowl (rice, chilli-con-carne and salads) and in a tall pot for the salsa. The two sides of the buffet were organised to avoid queuing at the buffet, but participants were able to go around freely (Fig 1) and there was not a predetermined participant flow. Participants had to serve themselves from the bowls, which were frequently replenished by the researchers. Participants were allowed to refill their plates as many times as they wished.

#### Priming

**Fig 2:**
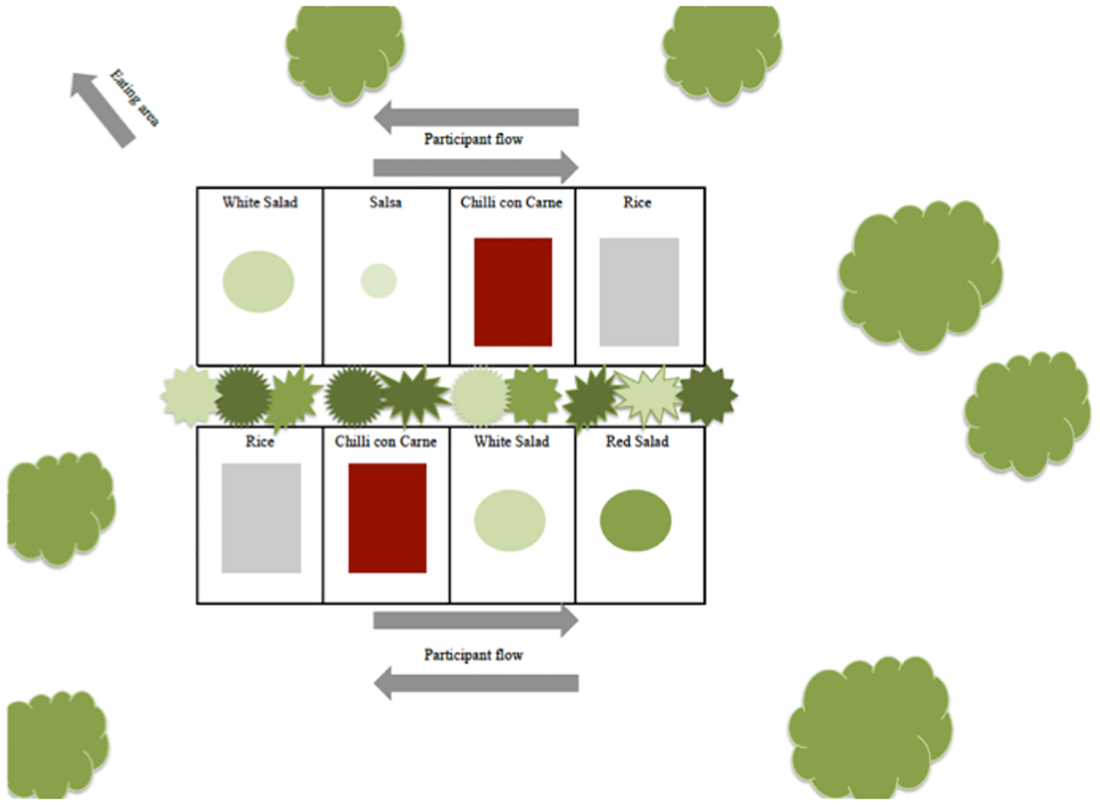
Visual presentation of the experimental setting: Priming. A green ambiance was created using plants and herbs.

The food environment was tailored to accommodate a green ambience of plants, green servings bowls and herbs in the dining area as shown in Fig 2. Plants were large green leafed and herbs included: basil, thyme, watercress and oregano. The plants and herbs were placed intentionally, so study participants would be visually exposed when interacting with the entire area I) at the cutlery section II) at the iBuffet III) at the dining tables IV) and as general décor in the eating area. Beside the visual effect, the fresh herbs provided a strong odour and participants could freely use the provided fresh herbs (basil, parsley) as part of their meal.

#### Default

**Fig 3:**
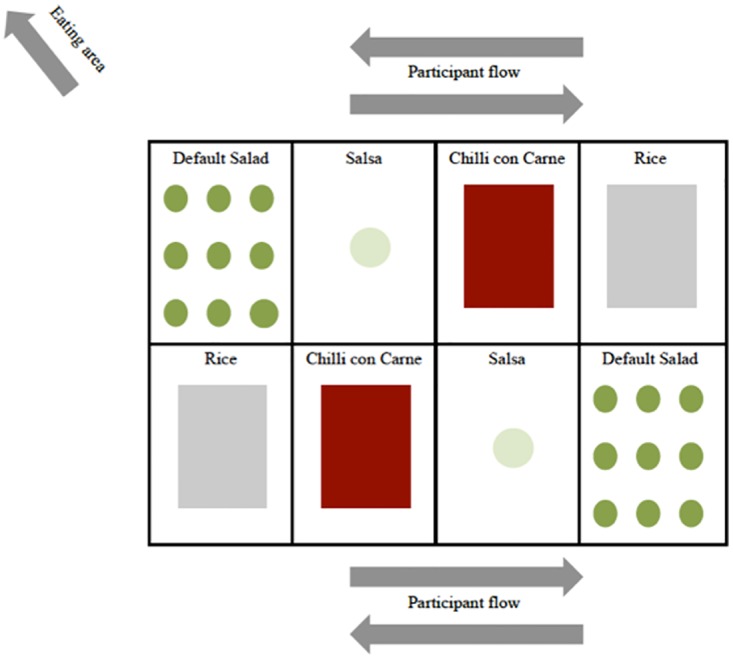
Visual presentation of the experimental setting: Default. The salad was pre-portioned into transparent bowls of 150g of the white salad and 50g of the red salad.

The two salads were divided into pre-portioned individual transparent ‘take-away’ bowls with the default amount of 150g of the white salad and 50g of the red salad. The bowls were placed at the iBuffet on serving area 4 and serving area 8 (see Fig 3). To replace where the white salad was on serving area 3, another salsa jar (containing the same salsa) was placed. Participants were to take the individual bowls with them, compared to the control situation where they had to serve themselves on their plates. The participants had the possibility of taking as many bowls of salad as they liked.

#### Perceived variety

**Fig 4:**
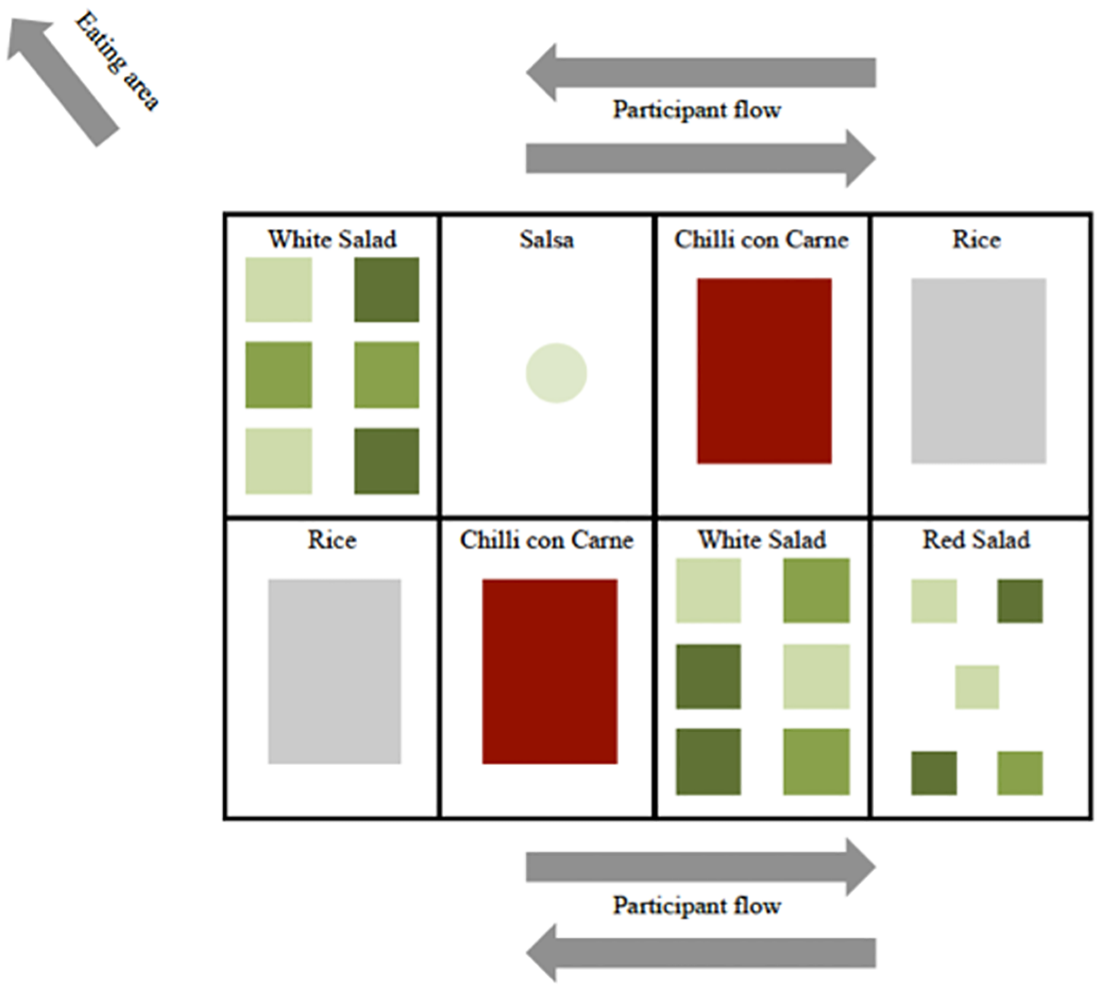
Visual presentation of the experiment setting: Perceived variety. The salad was split up to its component, increasing the visual variety and allowing participants to compose their own salad.

The two mixed salads served in the control setting were split into each of the ingredients in separate two-litre square bowls (Fig 4). All of the bowls were served on the same serving surface and the amounts served from each ingredient were accumulated into one measure of ‘White salad’ or ‘Red salad’. The overall selection of ingredients was exactly the same, but just divided in to more bowls, hence allowing each participant to compose their own salad.

### Data analysis

A complete case analysis was conducted where the difference in weighed intake (consumption minus wastage) between the two eating sessions was considered as a proxy for how well the Nudge worked.

A mixed general linear model analysis was performed to explore changes within the three nudges for rice, chilli con carne and total vegetable intake. The intake was set as the dependent variable, time as the fixed effect, subjects as the random effect and measures for appetite were included as co-variates. Q-Q plots and histograms were used to check for normality.

Oneway ANOVA with post hoc test was performed to explore any changes between the three nudges for: total intake, total chilli con carne intake, total rice intake and total vegetable intake. The intake difference in each food group was set as the dependent variable and the intervention was set as the fixed factor. Levene’s test of equality was non-significant, hence the assumption of equal variance was accepted.

The considered significant level was set at p≤0.05. Statistical analyses were performed with the software IBM SPSS Statistics version 22.0. Results are presented as means ± SDs, with corresponding p-values for comparisons for both the analysis within and between nudges.

## Ethics, consent and permissions

Participants were provided with information about VeggiEAT project (FP7–612326) and of the specific data collection that required them to attend two meals at the FoodScape Lab in convenient dates. All participants provided a written and signed consent to participate in the study prior to its start, and were free to leave at any point. No personal or sensitive information was obtained and data was/is stored according to Danish regulations, complying with EU Directive 95/46/EC on the protection of individuals with regard to the processing of personal data and on the free movement of such data. All required standards and criteria of The National Committee on Health Research Ethics under the Danish Ministry of Health have been met (http://www.dnvk.dk/) and complied with the Helsinki Declaration revised in 1983. The Scientific Committee of the Capital Region of Denmark revised the study protocol, gave the consent to go ahead with the study, and cleared from further formal approval (Ref. H-2-2013-FSP61).

## Results

### Participants

Table 1 presents the characteristics of participants divided by choice architecture exposure: priming, default and variety. In total, 222 participants were recruited. 110 were excluded, either because they did not meet the inclusion criteria or because they did not reply to further contact. After having completed one full test day, 24 of the 112 assigned participants did not show up for second session (21% drop out). Participant characteristics are given in Table 2. No major differences were detected between the basic characteristics of the participating sample in each of the three experiments, except in the level of education, where a difference was seen between Educational level I in priming and Educational level III in variety, however, education was not significant in the final modelling. The majority of the sample was composed of students (77%) where 67% of participants were in the process of a higher education (>4y) and 18% of the whole sample already had a higher education. There was no difference in the distribution of students between the three experiments according to gender, age, BMI or vegetable consumption.

**Table 1:** Characteristics of participants—Divided by choice architecture approaches: Priming, default and variety.

	Priming (n = 24)	Default (n = 33)	Variety (n = 31)	*p*^1^
**Women [n (%)]**	19 (70)	19 (58)	21 (66)	*ns*^2^
**Age (y)**	27.3±6.6^3^	25.9±7.1	26.3±6.5	*ns*
**BMI (kg/m**^**2**^**)**	23.4±5.6	22.6±2.6	22.5±2.8	*ns*
**Education level [n (%)]**				
*I*	9 (33)	20 (61)	21 (66)	0.02^4^*
*II*	10 (37)	7 (21)	10 (31)	
*III*	8 (30)	6 (18)	1 (3)	
**Self-reported daily intake of vegetables [g]**	320±170	276±179	343±338	*ns*

^1^ Between priming, default and variety by using One-way ANOVA, unless otherwise specified.

^2^ With the use of the chi-square test.

^3^ Mean ± SD, all such values.

^4^ With use of Fisher’s Exact test.

* There was a significant difference between educational level I in priming and educational level III in variety (p<0.01).

**Table 2:** Appetite score from control day and intervention day. Measurement scale used: 10 point Likert scale anchored from “I am not at all” (score: 1) to “I have never been more” (score: 10).

	Priming			Default			Variety		
	Control	Intervention	p*	Control	Intervention	p*	Control	Intervention	p*
**How hungry are you?**	7.5±1.3	6.3±1.7	*<0*.*01*	6.8±1.5	6.8±1.5	*ns*	6.8±1.4	6.4±1.6	*ns*
**How much do you think you can eat?**	7.7±1.2	6.7±1.1	*<0*.*01*	7.5±1.6	7±1.6	*0*.*08*	7.0±1.4	6.8±1.4	*ns*
**Can you eat more?**	3.2±2.2	3.3±1.8	*ns*	2.5±1.1	2.1±0.7	*0*.*04*	2.4±1.0	2.6±1.0	*ns*
**Are you satisfied?**	8.4± 1.3	8.1±1.6	*ns*	8.5±1.0	8.5±1.5	*ns*	8.6±1.0	8.4±1.0	*ns*

* Student t-test analysis of appetite scores from control day compared to intervention day

### Appetite score

Table 2 shows the appetite score from control day and intervention day measured by a 10 points scale (from “I am not at all hungry” = 0 to “I have never been more hungry” = 10). Priming was the only nudge group where a difference was found in the appetite score before eating. In the appetite score after eating, a difference was found in default for "Can you eat more?".

### Food intake

Table 3 shows the measured intake by in each of the experimental conditions and specified by food groups: vegetables, rice and chili con carne. Priming reduced the total food consumption by a mean of 168.87g (p<0.01) hereof mostly by decreasing the intake of chilli con carne (p<0.01). The default intervention successfully increased the intake of vegetables by a mean of 45g (p = 0.02). Providing a larger perceived variety at the buffet reduced the total intake of food by a mean of 99g (p = 0.02), mostly due to a decreased intake of chilli con carne (p<0.01). No effect was found in the intake of rice. Further analysis shows that for perceived variety gender had an effect on total intake, where men ate 201g less than the women in the group (p = 0.03). This is in contrast to priming where there was a tendency for men to eat 200g more than the women (p = 0.07). For the vegetable intake priming had an age-related effect with an 8g/year increase for the group (p = 0.01) (not tabulated).

**Table 3:** Intake in grams (g) divided by food group: Vegetables, rice and chilli con carne. Presented using means and SD. Test of significance using mixed modelling. Default n = 33, Priming n = 24, Perceived variety n = 31. “How hungry are you?” and “How much do you think you can eat?” were included as covariates in the analysis. “Can you eat more?” was further included as a covariate in the analysis for the Default nudge.

Nudge	Treatment	Vegetables (grams)			Rice (grams)			Chilli con Carne (grams)			Total intake (grams)			Total intake (kcal)		
		Mean	SD	*p*	Mean	SD	*p*	Mean	SD	*p*	Mean	SD	*p*	Mean	SD	*p*
Default	Control	193.67	82.61	0.016	152.39	103.50	0.76	275.33	145.12	0.41	621.39	240.99	0.33	596.30	264.04	0.62
	Intervention	238.88	141.49		156.97	102.90		257.79	148.78		653.64	229.82		615.13	258.45	
Priming	Control	214.58	75.83	0.15	145.33	80.30	0.27	278.38	147.92	<0.01	638.29	257.94	<0.01	602.03	267.16	<0.01
	Intervention	179.21	113.15		127.42	60.39		162.79	104.20		469.42	214.83		432.96	208.49	
Perceived Variety	Control	267.77	85.68	0.56	167.97	88.24	0.063	296.61	116.62	<0.01	732.36	240.32	0.019	678.64	248.21	<0.01
	Intervention	279.13	111.38		139.94	74.99		213.94	106.46		633.00	241.62		554.90	232.54	

Table 4 displays the observed energy intake differences specified by food groups. The quantities of vegetable consumed were statistically different between intervention type (F = 4.398 & p = 0.015). Comparing the effect size of the three nudges (presented as the difference between intervention and control in g) a significant difference was seen between priming and default, with a mean difference of 201g in total intake and 81g for total vegetable intake (Tables 3 and 4).

**Table 4:** Observed difference in energy intake (gram) session compared, divided by food group: Vegetables, rice and chilli con carne. Statistical test for significance of the three interventions compared to each other using one-way ANOVA with a post hoc test.

Nudge	Nudge comparison	Vegetables^1^ (grams)			Rice^2^ (grams)			Chilli con Carne^3^ (grams)			Total intake^4^ (grams)			Total intake^5^ (kcal)		
		Mean difference	SE	*p*	Mean difference	SE	*p*	Mean difference	SE	*p*	Mean difference	SE	*p*	Mean difference	SE	*p*
Default	Priming	80.59	29.29	0.027	22.49	22.53	0.61	98.04	32.95	0.015	201.12	58.87	<0.01	187.89	60.62	0.011
	Perceived Variety	33.86	27.31	0.47	32.61	21.00	0.31	65.13	30.72	0.11	131.60	54.89	0.062	142.57	56.52	0.047
Perceived Variety	Priming	46.73	29.69	0.29	-10.12	22.83	0.91	32.91	33.40	0.62	69.52	59.67	0.51	45.32	61.44	0.76

^1^ Observed power was 0.675.

^2^ Observed power was 0.267.

^3^ Observed power was 0.784.

^4^ Observed power was 0.887.

^5^ Observed power was 0.846

Looking at the energy intake, priming decreased the total energy intake by 169 kcal (p<0.01) and it decreased the energy from chilli con carne with 134 kcal (p<0.01). Default increased the energy intake from vegetables by 34 kcal (p<0.01) and offering perceived variety decreased the total energy intake by 124 kcal (p<0.01) and the chilli con carne with 96 kcal(p<0.01) (not tabulated). No effect was found in energy intake deriving from rice. The age-related effect seen for vegetable intake in grams was also seen in the energy analyses with a rise of 6 kcal per year increment (p<0.01).

ANOVA analysis with post hoc testing among the three nudges revealed significant differences in total energy intake between priming and default (Table 4). The 143 kcal difference in energy intake between perceived variety and default was significant (p<0.05), considering that the perceived variety intervention successfully decreased the energy consumption by 123 kcal (p<0.01). For energy intake deriving specifically from vegetables the default intervention increased the energy intake deriving from vegetables with 34 kcal (p<0.01). Additionally, the priming intervention achieved a non-significant decrease in energy from vegetables of 13 kcal, hence a net difference in energy intake from vegetables between the two experiments of 46 kcal (not tabulated).

## Discussion

The aim of this study was to test and compare three nudges in promoting vegetable consumption among test persons in a food lab-based experiment. The three employed nudges overall promoted what seem to be a healthier meal composition. However, the separate analysis for each experiment suggests that various automatic decision processes might be more effective depending on what the targeted food choice behaviour is. Priming attempted to prime vegetable choice, but significantly decreased the consumption of chilli con carne. A potential explanation could be found in the intense green ambience functioning as a food-external stimulus indirectly influencing the participant’s cognitive reactions [21]. This could lead to an activation of a reflective decision making, promoting a healthy-eater self-schema of food choice [40], hence not be an automatic process nudge, but a reflective. The visual presence of herbs and green ambience could remind the participants about a green healthy lifestyle and therefore activate behaviour relevant to his/her self-image, which would result in them limiting their intake of the unhealthy food. However, this study did not measure the participants’ level of health-schematic nor to what extent their food choice was reflective or not. Although self-reported appetite was significantly different in the intervention arm compared to its control in the priming strategy, it did not distort the effect of the nudge. Increasing vegetable consumption is a public health goal, which may contribute to dilute total energy intake and further contribute to tackling the obesity issues in EU. Even small changes towards a dietary pattern rich in foods of plant origin have positive effects on health in the longterm [41, 42]. Another finding of this study is related to the role of achieving satisfaction with less calorie dense foods. The questions on appetite (Table 2) provided interesting insights, particularly the fact that the differences in satisfaction and appetite levels were mainly not significant within and between participants, and if they achieved statistical significance, the difference was not meaningful.

The default intervention provided a fixed portion of salad as the status-quo or default bias, which successfully increased the intake of the salad, but did not decrease the total energy intake. The default portion was approximately 25% higher than the average intake during the control setting and the observed increase in intake of vegetables is in correspondence with what was anticipated according to the theory of plate clearing. Although the majority of research on default servings and plate clearing is conducted on actual plates [22, 23, 43, 44], our study showed similar effects although using a bowl-type container. The used bowl was transparent which might likewise have influenced the increase in intake as this has been illustrated to influence consumption volume [45].

The experiment of increasing the visual perceived variety decreased the self-served amount of meat and total energy, but the total intake of vegetables was not increased as hypothesised. Despite the experiment promoted a healthier meal composition, it was not supportive of the aim of this study. A non-conclusive gender-specific sub analysis did show, that the nudge might have increased women's intake whereas decreased men’s. It may be hypothesised for future studies, that the observed decrease found in men, may be due to the “cognitive burden” of choices between the ingredients in addition to the multitasking by handling the plate and checking-in at the iBuffet. In psychology studies it is shown that men are less efficient than women when multitasking and/or being in a choice situation [28, 46–48]. The causal link of these findings is based on the assumption, that the separation of fruit and vegetables components did in fact increase the visual variety. The actual variety stayed the same in control and interventions in order to be consistent with the definition of a nudge. Asking the participants of their perception of the vegetable variety would require them to embark in reflective decision-making thus losing the intended idea of nudging their food choice.

From a conceptual level an important concern to address is that default and variety conditions also differ in the easiness to reach the vegetables. More effortful cognitive tasks (choices) might alter the individual’s self-regulation capacity [49]. In the default condition participants had to simply grasp a pre-packed box while in the variety condition people had to self-serve and select themselves the amounts of vegetables they wanted, hence requiring more effort from their part. In a similar study [17] modification in the order of the buffet (vegetables first and hence easier to reach) and self-serving from different plates increased vegetable consumption while decreasing total energy intake without significantly altering the total quantity consumed. The present study was as close as possible to a real buffet setting where consumers are used to serve themselves, and shows a case where a promising successful nudge is one that made the healthier and desirable choices easier.

### Limitations and strengths

These experiments were held in, for some, a new and unfamiliar setting (the Foodscape Lab). During an unfamiliar eating session, the number of people may have had an impact on the generally low food consumption [50]. Likewise, participants knowing it was an experimental food setting (although not obvious at first) might have made them conscious about being measured and evaluated, leading to a decrease in intake [51]. This study only looked at a singular meal (lunch) and thus cannot conclude much about a whole day dietary intake; however, previous research suggests that participants did not compensate for their energy intake during lunch later in the day [52], hence indicating the potential for promoting health and well being through a singular lunch serving. This is underlined by the fact that an increasing number of meals are consumed outside home [53] and self-service buffet eating is associated with excess caloric intake [54, 55]. This current study had an over-representation of women compared to men, misrepresenting the national gender distribution of Denmark. The study had hoped to have a more representative sample, but it was not a requirement of the study’s design nor a theoretical basis for the alternative hypothesis’ expected effectiveness. Similar trends are seen in other Danish nutritional studies, with a general overrepresentation of women [38, 56, 57]. One reason for this is that women in general are more concerned with health and nutrition topics than men and therefore voluntarily sign up for studies of this nature [58, 59].

The major strength of this study was the iBuffet that with hidden weights measured exact amounts of food taken combined with the precise amount of food waste registered by the individual wristbands worn by the participants. This ensured a measure of the dietary intake and not retrospective intake with little or no inconvenience for the participating subject [60]. To the knowledge of the authors this is one of the few studies that has been performed in a setting that allowed complete accurate measurement of dietary intake, limiting bias from self-reporting [61, 62] and undetected plate waste.

## Practical implications & Conclusion

Considerable insight has been gained with regard to the complexity of the automaticities that influences food choice in a self-service setting. Nudging is being used as a theoretical and philosophical umbrella expression to understand these mechanisms. Mechanisms that are highly influential for shaping consumer behaviours in promoting healthier eating. Using nudging is a promising tool to shift the meal composition to a healthier one. This study has illustrated that serving vegetables in a default portion size is effective if the aim is to promote vegetable intake. If the aim is to decrease quantities of food eaten, this study suggest that priming vegetables and/or increasing the visual variety of vegetables could be effective.

With the global increase in out-of-home eating and the casual link with buffet style eating and lifestyle diseases, the public health community ought to address the issue of obesity in this context. Existing strategies to influence choices and behaviour have been successful to a certain extent, but not all of the way. This study illustrates that behaviour can be influenced and that healthy eating can be promoted solely based on the automatic decision-making system. Future research and public policy need to promote behaviours, acknowledging the reflective rational part of the consumer, as well as the habitual [63].

## Supporting information

S1 Dataset Datasets_2017_Article.sav.zip (ZIP)

## Abbreviations

BMI: Body Mass Index
cm: Centimetres
iBuffet: Intelligent Buffet
kg: Kilogram
NCD: non-communicable diseases
